# A toolbox for the comprehensive analysis of small volume human intestinal samples that can be used with gastrointestinal sampling capsules

**DOI:** 10.1038/s41598-021-86980-y

**Published:** 2021-04-14

**Authors:** Melany Rios-Morales, Mara P. H. van Trijp, Christiane Rösch, Ran An, Theo Boer, Albert Gerding, Naomi de Ruiter, Martijn Koehorst, M. Rebecca Heiner-Fokkema, Henk A. Schols, Dirk-Jan Reijngoud, Guido J. E. J. Hooiveld, Barbara M. Bakker

**Affiliations:** 1 Laboratory of Pediatrics, University of Groningen, University Medical Center Groningen, Antonius Deusinglaan 1, 9713 AV Groningen, The Netherlands; 2 Nutrition, Metabolism and Genomics Group, Division of Human Nutrition and Health, Wageningen University, Stippeneng 4, 6708 WG Wageningen, The Netherlands; 3 Laboratory of Food Chemistry, Wageningen University, Bornse Weilanden 9, 6708 WG Wageningen, The Netherlands; 4 Laboratory of Microbiology, Wageningen University, Stippeneng 4, 6708 WG Wageningen, The Netherlands; 5 Department of Laboratory Medicine, University of Groningen, University Medical Center Groningen, Hanzeplein 1, 9713 GZ Groningen, The Netherlands; 6 Laboratory of Pediatrics, University of Groningen, University Medical Center Groningen, PO Box 196, 9700 AD Groningen, The Netherlands

**Keywords:** Microbiology, Gastroenterology, Metabolomics, Dietary carbohydrates

## Abstract

Detailed knowledge on the fate of dietary components inside the human intestinal tract is lacking. Access to this inner world of digestion is now possible through novel human gastrointestinal sampling capsules. Due to the novelty of such devices, no methodology has been published to stabilise and analyse the resulting samples. A complicating factor is that excretion of such capsules in faeces may take days, while degradation of the dietary components continues. Therefore a stabilising reagent should be pre-loaded in the capsule to ensure the measurement of a representative sample. Considering the small volume of recovered samples, analytical methods must be optimized to collect as many data as possible from little material. We present a complete workflow for stabilising and analysing the fermentation status of dietary fibres in such samples, including microbiota, fibre degradation, and short chain fatty acids. The final quenching reagent was designed based on safety and effectiveness to inhibit fructo- and galacto-oligosaccharides degradation and short chain fatty acids production by human ileostomy microbiota, and subsequently validated in faecal samples. The final composition of the stock quenching reagent is 175 mM Tris, 525 mM NaCl, 35 mM EDTA, 12% SDS, and 8 M urea at pH 8.5.

Consumption of dietary fibres has been linked to many health benefits^1,2^. Many fibres can be fermented by gut microbiota, resulting in the production of short chain fatty acids (SCFA) and other metabolites^3^. Diet, and especially fibres, strongly affect the gut microbiota composition and metabolism. Distinctive microbiota profiles have been associated with healthy and diseased states^4^, including metabolic and intestinal disorders. One of the mechanisms of how microbiota affect host health is via the production of the SCFAs acetate, propionate, and butyrate^5,6^. In mice, the SCFA uptake into the host has been associated with the improvement of different metabolic markers such as insulin sensitivity^7^. In humans, most of our knowledge about microbial fermentation in the human gut derives from analysing stool samples, which are not necessarily representative for the content of the intestinal lumen since the gut environment changes throughout the complete length of the gut^8,9^. To study the gut-host interaction, we mostly rely on animal models, due to difficult access to the intestinal lumen in humans. Conventional methods of exploring and collecting human lumen samples remain invasive and include naso-intestinal catheters or colonoscopies^10^.

Recently non-invasive access to this inner world of gut microbiota and fermentation products has become possible with the development of novel human gastrointestinal capsules, some of which allow sampling of the luminal content^11–17^. Such devices allow a deeper understanding of diet–microbiota–host interactions. Nevertheless, due to the novelty of these devices, no associated analytical methodologies have been published. The current challenges are the small sampling volumes of around maximum 200 µL^14^, and the time delay between sampling and harvesting at body temperature since excretion of the capsule from the body can take up to days^12^. Therefore, at the moment of actuation, the sample needs to be stabilised to block further metabolism so that a representative sample is obtained. In order to do so, a suitable quenching reagent needs to be loaded in the capsule prior to swallowing. Because of the small maximum sample volumes, as much information as possible should be obtained from a single sample. This can be done by combining multiple analytical methods.

Here we present a toolbox comprising a complete workflow for analysing quenched intestinal samples from gastrointestinal sampling capsules. We developed a quenching reagent, which efficiently blocked microbial activity, i.e. fibre degradation, production of SCFA, and stabilised microbial DNA for 48 h at 37 °C. Bacterial fibre fermentation in the human intestine was mimicked by in vitro batch fermentations with different dietary fibres. Moreover, an efficient extraction procedure and workflow was developed to combine different analytical assays in the same small sample. Importantly, the safety of the quenching reagent for human use was taken into account. As a result, we obtained a toolbox of procedures to analyse a small representative intestinal sample obtained from gastrointestinal sampling capsules.

## Materials and methods

### Materials

Fibres used for small intestine (SI) samples were chicory inulin (degree of polymerization (DP) of 3–60; Frutafit HD, Sensus, Roosendaal, the Netherlands), chicory fructose-oligosaccharides (FOS, DP2-9; Frutalose OFP, Sensus), and galacto-oligosaccharides (GOS) powders composed of approximately 69% GOS and 28% mono- and disaccharides (Vivinal GOS, FrieslandCampina, Wageningen, the Netherlands). For faecal samples, fibres used were FOS (DP2-9; Frutalose OFP, Sensus) and GOS (Vivinal, FrieslandCampina). The commercial enzymes glycoside hydrolase enzyme β-galactosidase (EC 3.2.1.23)^18^ isolated from Aspergillus oryzae (Lactase DS-K, Amano Enzyme Inc., Japan), and endo-inulinase (EC3.2.1.7) isolated from Aspergillus niger^19^ (Novozym 960, Novozymes A/S, Denmark) were used. SCFA were used as a sodium salts (sodium acetate, sodium propionate and sodium butyrate), and were all obtained from Sigma Aldrich (Missouri, USA). All other chemicals used were at least of (bio)chemical grade.

### Methods

#### Conditioning of human small intestine samples

Ileostomy effluents from five male and female Caucasian volunteers were collected from the distal ileum, as previously described^20^. The subjects had not been treated with antibiotics, pre- or probiotics for at least 3 months directly before effluent donation. The ileostomy effluent was collected after a 14 h fasting period in the morning, and kept at − 20 °C to minimize bacterial activity until further use within 9 h. Effluents were diluted to a 20% (w/v) slurry in standard ileal efflux medium (SIEM)^21^ at pH 7.0. The SIEM was adapted as described elsewhere^22^, but without Tween 80 to avoid interference with high performance anion exchange chromatography with pulsed amperometric detection (HPAEC-PAD), with less carbohydrates namely a mixture of pectins, xylan, arabinogalactan, amylopectin and starch (in total 0.24 mg/mL), and with MgSO_4_ (0.8 mg/mL)^20^. The diluted ileostomy effluents were sieved (1.6 mm sieve) to get rid of large food particles, and afterwards directly used for preselection of the ileostomy effluent in SIEM for 15 h under anoxic conditions (81% N_2_, 15% CO_2_ and 4% H_2_, at 37 °C, shaking at 100 rpm)^23^, for removal of left over carbohydrates. The preselected sample was incubated in SIEM containing the selected dietary fibres for conditioning. This resulted in a final 10% slurry (w/v) of the SI sample and 10 g/L dietary fibres, namely inulin and FOS in a 1:1 w/w ratio. Conditioning incubation took also place under anoxic conditions at 37 °C, shaking at 100 rpm for 5 h. Subsequently, the content of the fermentation bottles was transferred to sterile tubes, frozen in liquid nitrogen, and stored at − 80 °C till further experiments.

#### Conditioning of human faecal samples

Faecal samples from four healthy, adult female and male human volunteers were collected, and stored at − 80 °C till further use. The faecal samples were mixed and diluted in SIEM with fibres at pH 6.0, obtaining for each faecal sample a 8% faecal slurry (w/v) and 10 g/L dietary fibres. Conditioning incubation took place under anoxic conditions at 37 °C, shaking at 100 rpm for 30 min to avoid complete fibre degradation. Afterwards, the content of the fermentation bottles was transferred to sterile tubes, frozen in liquid nitrogen, and stored at − 80 °C till further experiments.

#### Composition of the quenching reagent

The final composition of the stock quenching reagent after optimisation was: 175 mM Tris, 525 mM NaCl, 35 mM EDTA, 12% SDS, and 8 M urea at pH 8.5. The quenching reagent solution was heated at 37 °C until clear. The quenching reagent was freshly prepared at the day of experiments. In the fermentation samples the quenching reagent was used in a 1:5 v/v ratio, resulting in a final concentration of the components in the mixture of 35 mM Tris, 105 mM NaCl, 7 mM EDTA, 2.4% SDS, and 1.6 M urea.

#### Estimation of interference and efficacy of the quenching reagent

To test the interference of the quenching reagent and its individual components (urea, NaCl, SDS) with the analytical methods, the quenching reagent or its components were separately added to standard mixtures of fibres and SCFA. For fibres, 50 μL quenching reagent was added to 200 μL of standard mixtures of 5 mg/mL fibres in water. The mixture of quenching reagent with fibres was diluted in water to a final concentration of 200 µg/mL fibres in the sample before oligosaccharide analysis. For SCFA analysis, 100 μL of quenching reagent or urea, NaCl, and SDS diluted in water (1:5 v/v ratio), were added to a mixture of SCFA (between 0 and 20 mM). For precipitation of the SDS, different volumes (32, 65, 130 μL) of 4 M KCl were added on ice to the mixture and centrifuged before organic solvent extraction was performed.

To test the efficacy of the quenching reagent, incubations were started using conditioned faecal samples (“Conditioning of human faecal samples” section). In an Eppendorf tube, either 50 µL of PBS (as control) or 50 µL of quenching reagent was added to 200 µL of conditioned fermentation samples. The tube was flushed with nitrogen gas to create an anoxic environment. The mixtures were incubated for 48 h at 37 °C with shaking at 400 rpm. The incubations were stopped by freezing at − 80 °C (Supplementary Fig. S1). Reference samples at time 0 h were frozen at − 80 °C directly for comparison.

#### Analysis of short chain fatty acids

Samples (100 µL) were thawed and the following additions were made in the order of: 400 µL of PBS, 100 µL of 0.5 mg/mL 2-ethylbutyric acid solution (internal standard for SCFA measurements, the same stock of internal standard was used for calibration curves and samples within the same run), 20 µL of 20% 5-sulfosalicylic acid^24^. SDS in the quenched samples was precipitated by adding 32 µL of 4 M KCl and keeping the samples on ice (molar concentration ratio of 17:1 of KCl over SDS in the final sample). Next, samples were homogenized by bead beating for 30 s at 5000 rpm with 4–5 2.3 mm zirconium beads (Precellys 24, Bertin Technologies, Montigny Le Bretonneux, France) at 4 °C, after which the samples were centrifuged (20 min, 15,000 × *g*, 4 °C) and the supernatant was transferred to a glass vial. To the supernatant, a spatula tip of solid NaCl and 2 mL diethylether were added. Tubes were vortexed for 10 min at 4 °C and centrifuged (10 min, 1200 g, 4 °C). To 500 µL of the organic layer, 50 µL of N-tert-butyldimethylsilyl-N-methyltrifluoroacetamide (MTBSTFA) was added for overnight SCFA derivatization in a glass vial. The remaining aqueous phase was stored at − 80 °C for oligosaccharide analysis. Concentrations of the different SCFA were measured using an Agilent 5975C series gas chromatography/mass spectrometry (GC–MS) (Agilent Technologies, Santa Clara, USA). The GC was equipped with a ZB-1 column (Phenomenex, Torrance, USA). Mass spectrometry analysis was performed by positive electron ionization. Ions monitored were m/z 117 for acetate, m/z 131 for propionate, m/z 145 for butyrate and m/z 173 for 2-ethylbutyric acid^24^.

#### Analysis of the oligosaccharide composition

The obtained aqueous phase after SCFA extraction was centrifuged 10 min at RT at 15,000 × *g*. 200 µL were used directly for analysis of mono-, di-, and oligosaccharide profiles of GOS, FOS, and inulin, by HPAEC-PAD (Dionex Corporation, Sunnyvale, CA, USA). The system, columns, elution gradient, and flow rate were used as described elsewhere^25^.

#### Determination of microbiota composition

##### DNA extraction

100 µL of the fermentation sample was used for DNA extraction. Cell lysis was achieved by a repeated bead beating technique^26^, in combination with ASL Stool lysis buffer (Qiagen, Hilden, Germany). The obtained lysate (supernatant) was stored at − 20 °C, and used for DNA extraction. First, AL buffer (Qiagen) was added to the lysate, in the ratio 200 µL AL buffer to 250 µL lysate, and afterwards DNA was extracted and purified using the QIAamp DNA Mini Kit (Qiagen).

##### Total bacterial 16S rRNA gene copy number quantification

The total bacterial abundance in the pellet versus the supernatant was quantified by amplifying a conserved region of the 16S rRNA gene in a CFX384 Real-Time PCR detection system (Bio-Rad Laboratories, Hercules, USA). gDNA was diluted to 5 ng/µL. The primers and PCR cycling conditions were described elsewhere^27^.

##### Microbiota composition

Microbiota composition was determined via sequencing of the variable V4 region of the 16S rRNA gene. Triplicate PCR reactions were performed in 35 µL, containing 7 µL 5 × Phusion Green HF buffer, 0.7 µL 10 mM dNTPs (Promega, Madison, USA), 0.4 µL Phusion hot start II DNA polymerase (2 U/µL), 25.5 µL nuclease-free water, 0.7 µL of extracted template DNA (20 ng/µL) and 0.7 µL of each of the barcoded primers 515F^28^ and 806R^29^ (10 µM)^30^. Cycling conditions were as follows: 98 °C 30 s, 25 cycles of 98 °C 10 s, 50 °C 10 s, 72 °C 10 s, and 72 °C for 7 min. Pooled PCR products were checked on a 1.3% agarose gel, and purified using magnetic beads (MagBio Genomics Inc., Gaithersburg, USA). PCR product concentrations were measured using Qubit dsDNA BR buffer and dye (Invitrogen, California, USA), on a DS-11 FX fluorometer (DeNovix, Wilmington, USA). Afterwards, a library containing an equimolar mix (200 ng each) of purified PCR products was prepared. The resultant library was concentrated by using magnetic beads, and sequenced on the Illumina HiSeq2500 platform (Eurofins GATC Biotech, Konstanz, Germany). Raw sequencing data was processed using NG-Tax analysis pipeline version 1.0 with default settings^31,32^. Reads were selected with perfect matching primer sequences and de-multiplexed by selecting read pairs with perfectly matching valid barcodes. Amplicon sequence variants (ASV) were picked as follows: sequences were ordered by abundance per sample and reads were considered valid when their cumulative abundance was ≥ 0.1%. Taxonomy was assigned using the SILVA database (version 128), with a confidence of > 80% for genus level classification. For microbiota composition analysis R version 3.5.1. was used. Microbiota composition at time point 0 and 48 h were correlated using the relative abundances on genus level with Pearson correlations.

### Statistical analysis

The same individuals were present in both the control and quenching reagent group (related groups). Distribution was checked with the Shapiro–Wilk Normality Test. Statistical analysis on % fibre remainders at the 48 h time point in the faecal samples was performed using independent t-tests for a non-normal distribution. *P* values of < 0.05 were considered statistically significant. Statistical analyses were performed using R version 3.5.1.

### Ethics approval and consent to participate

The Medical Ethical Reviewing Committee of Wageningen University (METC-WU) has evaluated this study and concluded that research in which subjects are not physically involved and anonymously donate stools, does not require ethical approval from a recognized medical ethics committee. The subjects gave oral consent for the use of their faeces or ileostomy effluent in the in vitro experiments. All specimens were used and coded anonymously.

## Results

### Quenching reagent development

#### Incubations with commercial carbohydrate degrading enzymes and fibres

When gastrointestinal sampling capsules are used to study microbial metabolism of fibres in the gut in vivo, there is a considerable time delay between sampling and retrieving the capsule after defecation. Therefore, microbial metabolism should be stopped immediately after sampling, while at the same time the quenching solution should not decompose the fibre. The presence of a quenching solution inside sampling devices becomes essential. After several unsuccessful attempts to develop a quenching fluid based on their potential to inhibit fibre degrading enzymes (Supplementary Table S1), a commonly used bacterial lysis buffer was adopted as quenching reagent (NaCl, EDTA, Tris, SDS, pH 8.5)^33^, to which urea was added as general protein denaturant^34^. This quenching reagent was first tested for its effectiveness to inhibit the degradation of GOS and chicory FOS/inulin by commercially available β-galactosidase and endo-inulinase, respectively (Supplementary Table S2 and Supplementary Fig. S2). Furthermore, the effect of metal ions in water, Ag^+^, Cu^2+^, Zn^2+^, and Ag^+^/Cu^2+^ were included because of their known inhibitory effect in enzymatic fibre degradation^35,36^. Ag^+^ inhibited GOS breakdown but did not completely inhibit FOS/inulin breakdown by their corresponding enzymes (Supplementary Table S1). The enhanced bacterial lysis buffer almost abolished GOS and FOS/inulin degradation as is clear from the comparison of the DP profiles after incubation with those of the initial substrates (Supplementary Table S2, Supplementary Fig. S2). Subsequent addition of Ag^+^ to the quenching reagent effectively inhibited GOS degradation completely (Supplementary Fig. S2A, B), whereas supplementation of the quenching reagent with proteinase K appeared to be ineffective for inhibiting fibre degradation.

Next, the effectiveness of the quenching reagent set at different pH values was evaluated compared to degradation in water (Supplementary Fig. S2C, D), to see whether a pH outside the optimal enzyme pH activity range (4.0–9.0^37–40^) improved effectiveness. At pH 6.5 enzymatic FOS/inulin breakdown was completely inhibited, while GOS breakdown was only partially inhibited. At pH 9.5 both GOS and FOS/inulin breakdown were completely inhibited. When compared to the degradation of both type of fibres in quenching reagent at the normal pH 8.5, inhibition of degradation at pH 9.5 was very similar to that at pH 8.5 (Supplementary Fig. S2A versus S2C, and Fig. S2B with S2D). Therefore, taken into account the pH sensitivity of DNA^41^, the pH of the quenching reagent was set at 8.5^42^. Combining all the preliminary results we continued testing with the quenching reagent containing 50 mM NaCl, 10 mM EDTA, 1.5% SDS, 8 M urea, 50 mM Tris at pH 8.5 with or without 20 mM Ag^+^.

#### Evaluation of fibre breakdown quenching in human small intestine fermentation samples

**Figure 1:**
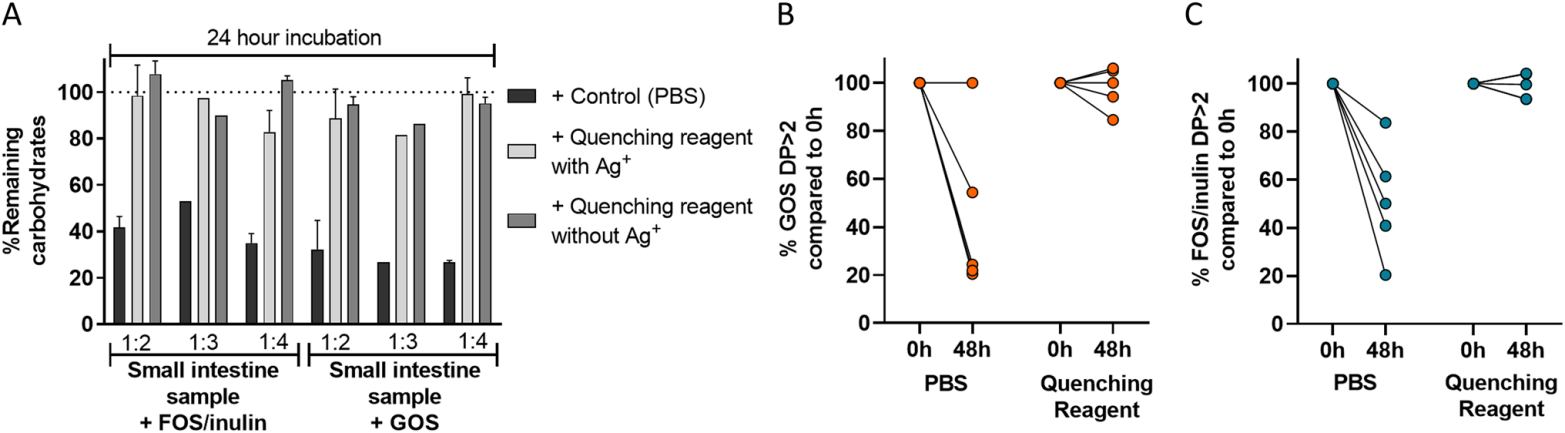
Quenching reagent effectiveness in small intestine samples. (**A**) The quenching reagent stock solution (pH 8.5, 50 mM Tris, 150 mM NaCl, 10 mM EDTA, 1.5% SDS, 8 M urea) with/without Ag^+^ was tested on chicory FOS/inulin and GOS breakdown by one human small intestine sample in vitro in a 24 h incubation. The tested ratios of quenching reagent:fermentation sample were 1:2, 1:3, 1:4 v/v. Fermentation samples were centrifuged and the supernatant was diluted in water, so the maximum fibre concentration became 0.5 mg/mL, and analyzed by HPAEC-PAD. (**B**, **C**) The percentage of GOS DP > 2 (**B**) or FOS/inulin DP > 2 (**C**) after 48 h in vitro fermentation by five human small intestine samples in the presence of quenching reagent (pH 8.5, 175 mM Tris, 525 mM NaCl, 35 mM EDTA, 12% SDS, and 8 M urea) or control, 1:5 v/v. Each line represents one individual. Breakdown was expressed as % remaining carbohydrates after incubation versus the initial carbohydrates present at 0 h. *GOS* galacto-oligosaccharides, *FOS* fructo-oligosaccharides.

For in vivo experiments in humans, gastrointestinal sampling capsules have to be preloaded with a quenching reagent. To mimic the in vivo situation, the effectiveness of the quenching reagent to inhibit fibre breakdown was studied in vitro in ileostomy samples, representative for small intestine samples at various dilution ratios of the quenching reagent. Fibres (chicory FOS/inulin or GOS) were incubated with ileostomy samples for 24 h at volume ratios of quenching fluid over ileostomy samples of 1:2, 1:3 and 1:4 (v/v) with or without 20 mM Ag^+^ (Fig. 1A). At all ratios tested, fibre degradation was almost completely inhibited, irrespective of the presence of Ag^+^. Therefore, Ag^+^ was further omitted from the quenching reagent. The components in the quenching reagent were concentrated to achieve effective quenching also in other volume ratios, therefore we continued testing with the quenching reagent containing 175 mM Tris, 525 mM NaCl, 35 mM EDTA, 12% SDS, and 8 M urea, with pH 8.5 in the stock solution. Degradation was minimal over 48 h incubation of both GOS and FOS/inulin in the presence of this quenching reagent (Fig. 1B, C) at a ratio of quenching reagent of 1:5 v/v in ileostomy samples from five different subjects (97.9% ± 7.9% GOS; 99.0% ± 4.8% FOS/inulin), compared to PBS control (30.3% ± 14% GOS; 51.2% ± 21% FOS/inulin). Overall, the quenching reagent at pH 8.5 effectively blocked GOS and FOS/inulin degradation in ileostomy samples at a ratio of 1:5 v/v.

### Interference of the quenching reagent and its components in analytical protocols

The interference of the quenching reagent and its components in the analytical techniques were evaluated next.

#### Interference of the quenching reagent in SCFA analysis

**Figure 2:**
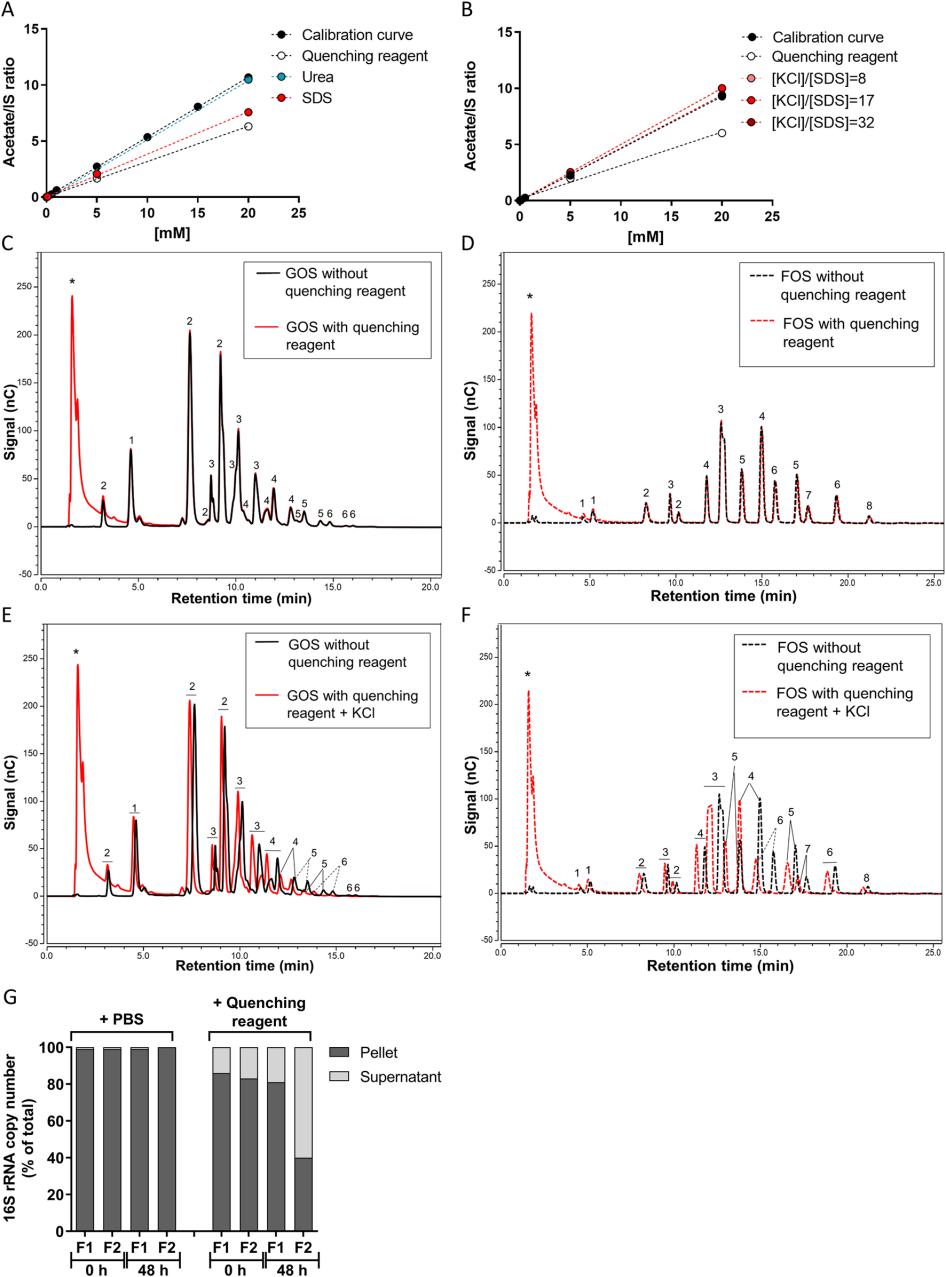
Quenching reagent interference in SCFA, oligosaccharides and bacterial 16S rRNA copy number analysis. (**A**) Calibration curves made of acetate concentrations in PBS in the presence of quenching reagent or its major components. (**B**) Calibration curve of acetate after precipitation of SDS with KCl in different KCl/SDS molar ratios. HPAEC chromatograms of GOS (**C**, **E**) and FOS (**D**, **F**) prepared in water. The black lines represent the mixture of fibre in water, and the red lines represent the fibre in water in the presence of quenching reagent (**C**, **D**) or in the presence of quenching reagent after precipitation of SDS with KCl (**E**, **F**). The numbers in the chromatograms represent the degree of polymerization, 1 = monomers, 2 = dimers, ≥ 3 = oligomers, and * are components present in the quenching reagent. (**G**) The % 16S rRNA bacteria copy number present in the supernatant or in the pellet versus total from two faecal fermentation samples were calculated in the presence of quenching reagent or PBS at 0 and 48 h. The total is the 16S rRNA copy number in pellet plus supernatant.

After we optimized the analysis of SCFA for intestinal and faecal samples^24^ (Supplementary Fig. S3), we tested whether the quenching reagent affected the extraction and the performance of the SCFA analysis (Fig. 2A, B). SCFA standard solutions were analysed in the presence or absence of the quenching reagent or its separate components by GC–MS. The quenching reagent led to an underestimation of SCFA concentrations, and SDS was found to be the interfering component (Fig. 2A and Supplementary Fig. S4A, C). Therefore, SDS was precipitated on ice by the addition of various amounts of KCl prior to SCFA extraction (Fig. 2B and Supplementary Fig. S4B, D). At a molar concentration ratio of 17:1 of KCl over SDS (1 M of KCl over 0.06 M of SDS in the mixture) SCFA were fully recovered as compared to the analysis in water. Precipitation of SDS by the addition of KCl on ice was effective preventing SDS interfering in the analysis of SCFA.

#### Interference of the quenching reagent in oligosaccharide analysis

The effect of SDS and its precipitation was also studied on the analysis of fibre by HPAEC-PAD. Standard mixtures of GOS and FOS in water were prepared without quenching reagent, with quenching reagent, and with quenching reagent with SDS removed using KCl precipitation and analysed. The quenching reagent per se did not affect the analysis of fibre standards (Fig. 2C, D). The combined addition of the quenching reagent and KCl influenced the retention time of the mono- di- and oligomers (Fig. 2E, F), but did not affect the signal response for the various compounds in the FOS and GOS mixtures nor the total peak area (Supplementary Table S3). Elution from the column shifted to more early retention times, probably caused by chloride ions (anions) from KCl. For correct peak identification, the quenching solution and KCl were also added to oligosaccharide standards from hereon.

#### Interference of the quenching reagent on the recovery of bacterial DNA

Commonly the pellet obtained from samples with microbial content by centrifugation is used for bacterial DNA extraction. However, since the quenching reagent is a lysing reagent, we investigated if the bacterial DNA was fully recovered in the pellet. To this end, two faecal samples were incubated for 48 h in the presence of quenching reagent or PBS. Afterwards, the pellet was separated from the supernatant by centrifugation (15 min, 4 °C, 18,000 × *g*). Subsequently, DNA was extracted from the pellet and from the supernatant. For both faecal samples, in the presence of quenching reagent a substantial part of the 16S rRNA bacterial copy number was found in the supernatant at 0 h and 48 h, compared to 0–1% in the PBS controls (Fig. 2G). Therefore, an aliquot of the intact homogenized sample must be used for DNA extraction, rather than only the pelleted sample.

### Validation of the quenching reagent efficiency in in vitro incubations with human faecal microbiota

Now that we developed a quenching reagent and a protocol to analyse fibre degradation, bacterial composition and SCFA concentrations in quenched samples, the protocol was tested in faecal samples, a more challenging matrix with respect to fibre fermentation. To this end, four human faecal samples were inoculated in in vitro batch fermentations of GOS and chicory FOS.

#### Quenching of oligosaccharides degradation and SCFA appearances in faecal samples

**Figure 3:**
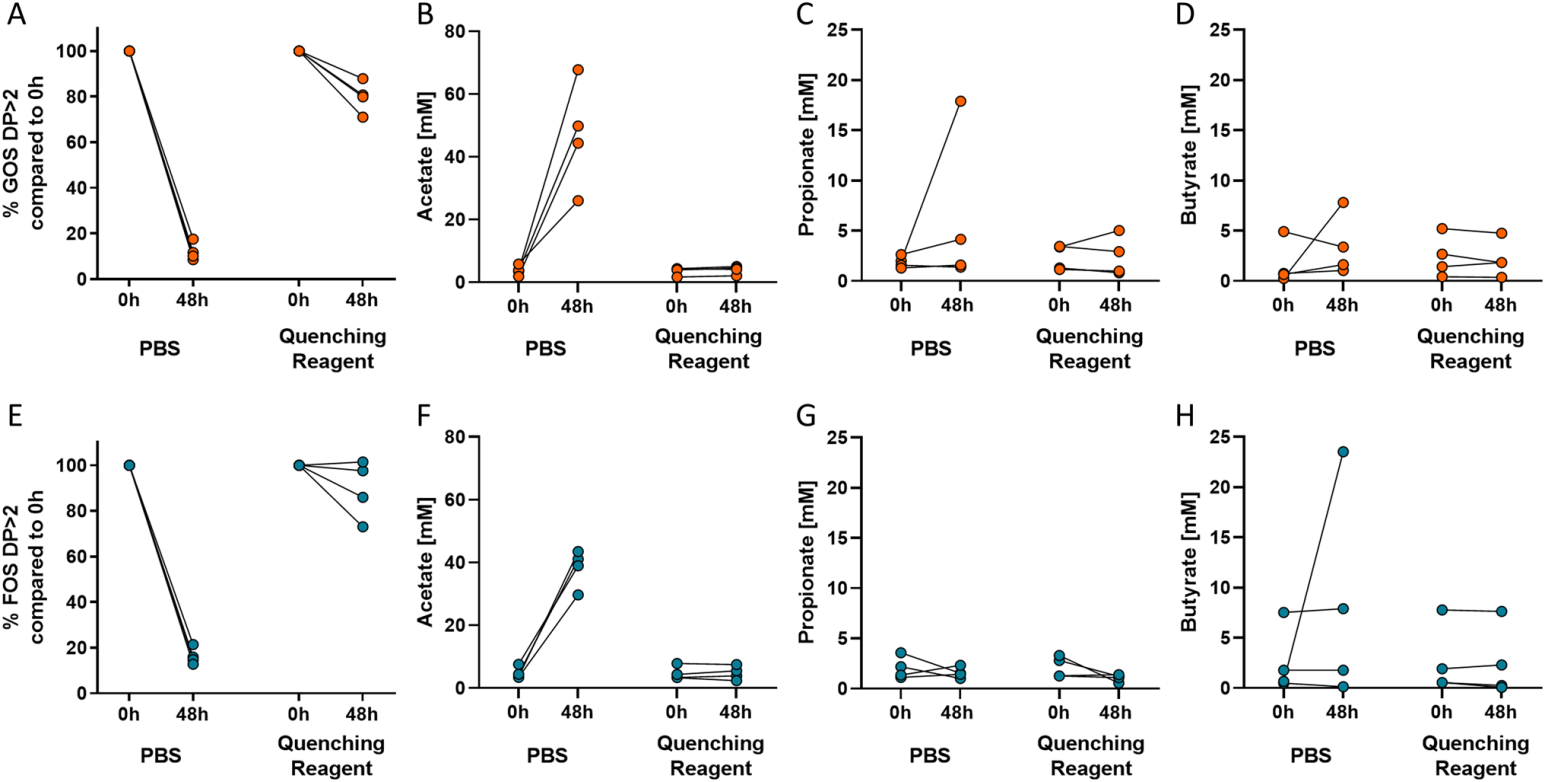
Quenching reagent effectiveness in faecal samples. The percentage of GOS DP > 2 (**A**) or FOS DP > 2 (**E**), and concentrations of SCFA in fermentations of GOS DP > 2 (**B**–**D**), or FOS DP > 2 (**F**–**H**) from four human faecal samples after 48 h of in vitro incubation versus the start of the incubation (0 h). Each line represents one individual. Fibres breakdown was expressed as % remaining carbohydrates after incubation versus the initial carbohydrates present at 0 h. *FOS* fructo-oligosaccharides, *GOS* galacto-oligosaccharides.

Oligosaccharide analysis showed that in incubations without the quenching reagent (PBS controls) less than 18% GOS and less than 21% chicory FOS was left after 48 h (Fig. 3A, E). Addition of the quenching reagent at a ratio of 1:5 v/v preserved on average 80.0% ± 6.0% of the GOS, and on average 89.5% ± 11.1% of chicory FOS during 48 h incubations, significantly more than in PBS controls (*P* < 0.05).

During 48 h incubation in the presence of the quenching reagent, SCFA production and interconversion was completely blocked as can be concluded from the constant SCFA concentrations compared to 0 h (Fig. 3B–D, F–H). In the absence of the quenching reagent, the SCFA concentrations increased substantially over 48 h.

#### Quenching of bacterial 16S rRNA copy number and microbiota composition in faecal samples

**Figure 4:**
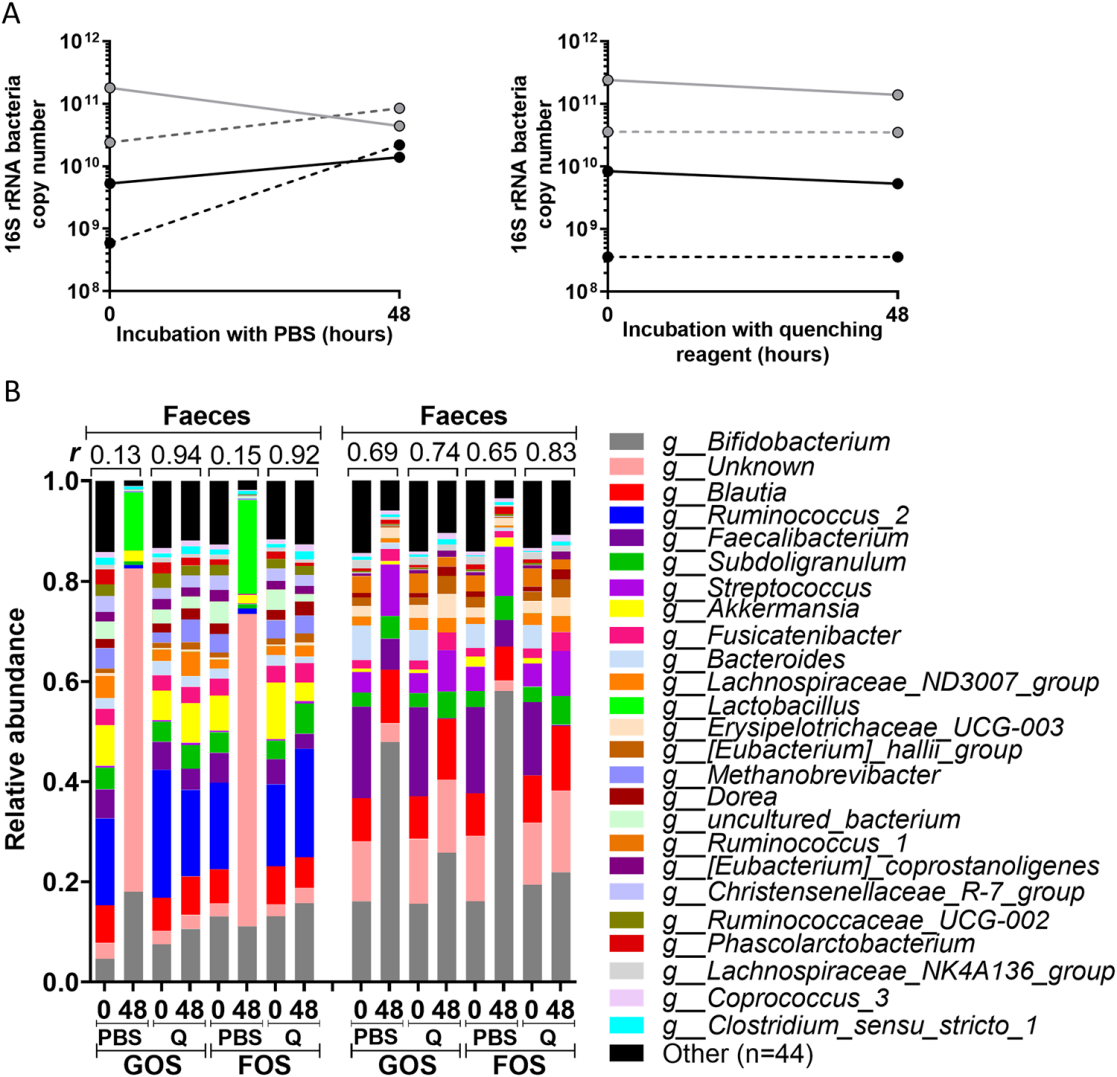
Quenching of the 16S rRNA bacteria copy numbers and microbiota composition in human faecal samples. (**A**) The total bacteria 16S rRNA copy numbers in the faecal fermentation samples, that were mixed and diluted in SIEM medium with fibres, in the presence of quenching reagent or PBS at 0 and 48 h. The same colour represents faeces from the same individual. (**B**) The relative microbiota composition at 0 h and 48 h after addition of PBS or quenching reagent ‘Q’ in conditions with added GOS or chicory FOS for two faecal donors. The top 25 genera present in the dataset are shown. Pearson correlation coefficients were calculated on the relative abundances at genus level, and shown in the graph as correlation number. *Q* quenching reagent, *FOS* fructo-oligosaccharides, *GOS* galacto-oligosaccharides.

16S rRNA bacterial copy number is used as indication for the total number of bacteria. In the PBS incubations, the total 16S rRNA bacterial copy number increased substantially over 48 h, except for one faecal sample, demonstrating bacterial proliferation over time (Fig. 4A), likely due to the presence of undigested material in the faecal sample. In one faecal fermentation the 16S rRNA copy number decreased, which may due to e.g. substrate depletion and accumulation of (toxic) products. Nevertheless, in all samples where the quenching reagent was added at a ratio of 1:5 v/v, the total 16S rRNA copy number did not change, indicating effectively blocking biomass changes (Fig. 4A). The microbiota composition was determined in two faecal samples for GOS and chicory FOS fermentation at 0 and 48 h (Fig. 4B). In the PBS conditions the microbiota composition changed between 0 and 48 h, as indicated by the correlation coefficients of the relative microbiota composition at the genus level, which were < 0.15 and < 0.69 for two faecal donors. When quenching reagent was added at a ratio of 1:5 v/v, the 48 h microbiota mimicked the microbiota present at the start, as indicated by the correlation coefficients of the relative microbiota composition at the genus level between 0 and 48 h, which were > 0.92 and > 0.74. In conclusion, addition of the quenching reagent did not only preserve the bacterial 16S rRNA copy number, but also the microbial composition.

### Development of a toolbox to measure fibre fermentation in very small gastrointestinal samples

**Figure 5:**
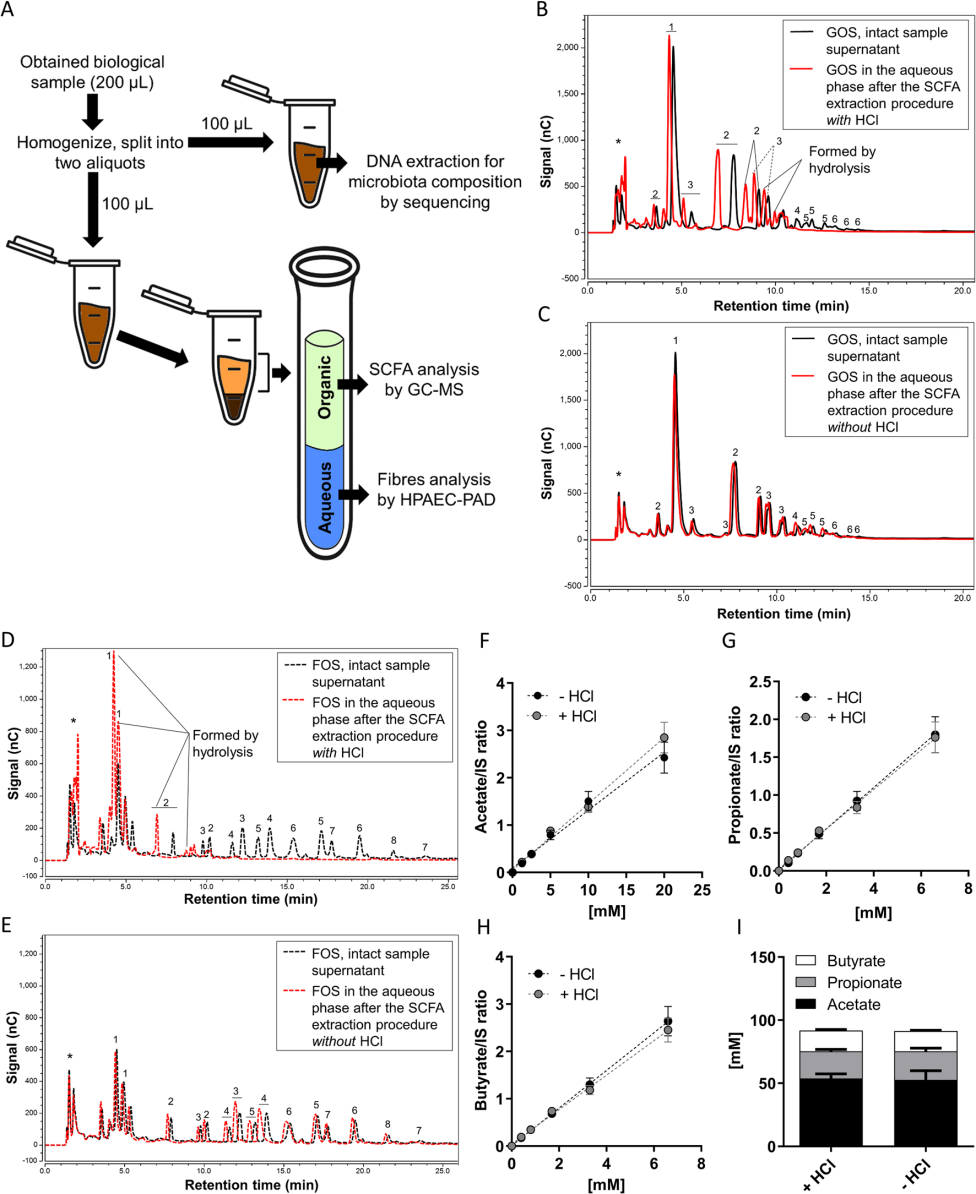
Combined protocol of fibres and SCFA analysis. (**A**) A schematic workflow for the combined analysis of a very small gastrointestinal sample volume. HPAEC chromatograms of GOS faecal fermentation samples (**B**, **C**) and chicory FOS faecal fermentation samples (**D**, **E**). The black lines represent fibres present in the intact sample supernatant, and the red lines represent fibres present in the aqueous phase after the SCFA extraction procedure with HCl (**B**, **D**) or without HCl (**C**, **E**). The numbers in the chromatograms represent the degree of polymerization, 1 = monomers, 2 = dimers, ≥ 3 = oligomers, and * are components present in the quenching reagent and in the medium. The calibration curves of concentrations of acetate (**F**), propionate (**G**) and butyrate (**H**) with and without the addition of HCl in the extraction protocol, and SCFA concentrations (**I**) in the same faecal sample with and without the addition of HCl (mean ± SEM, n = 3–5) . *GC–MS* gas chromatography–mass spectrometry, *HPAEC-PAD* high performance anion exchange chromatography with pulsed amperometric detection.

Volumes of gastrointestinal samples, obtained from gastrointestinal sampling devices, are maximally 200 µL^14^. To obtain as much information as possible, we developed a workflow in which analyses were combined to allow the most efficient workup of the samples. In the final workflow (Fig. 5A), prior to extraction of SCFA and oligosaccharides an aliquot of the intact homogenized sample was taken for microbiota analysis. For fibre and SCFA analysis, the analytical protocols were optimized to avoid splitting the sample before extraction.

With extraction of SCFA with organic solvents after acidification of the sample using HCl^24^, the hydrophilic oligosaccharides (GOS and chicory FOS) were expected to remain in the aqueous layer (Fig. 5A). Therefore, the recovery of GOS and chicory FOS from the aqueous phase after SCFA extraction was compared to that from the intact sample (Fig. 5B–E). We observed that in the aqueous layer the oligosaccharides in GOS and FOS were lost (Fig. 5B, D), and mostly hydrolysed into monomeric units. The SCFA extraction procedure caused hydrolysis of GOS and chicory FOS, most likely because the samples were acidified by addition of HCl prior to SCFA extraction. When HCl was omitted from the sample treatment, acid hydrolysis of GOS and chicory FOS was prevented, and the fibres were completely recovered in the aqueous phase (Fig. 5C, E). The extraction procedure only caused a slight shift in retention time.

**Figure 6:**
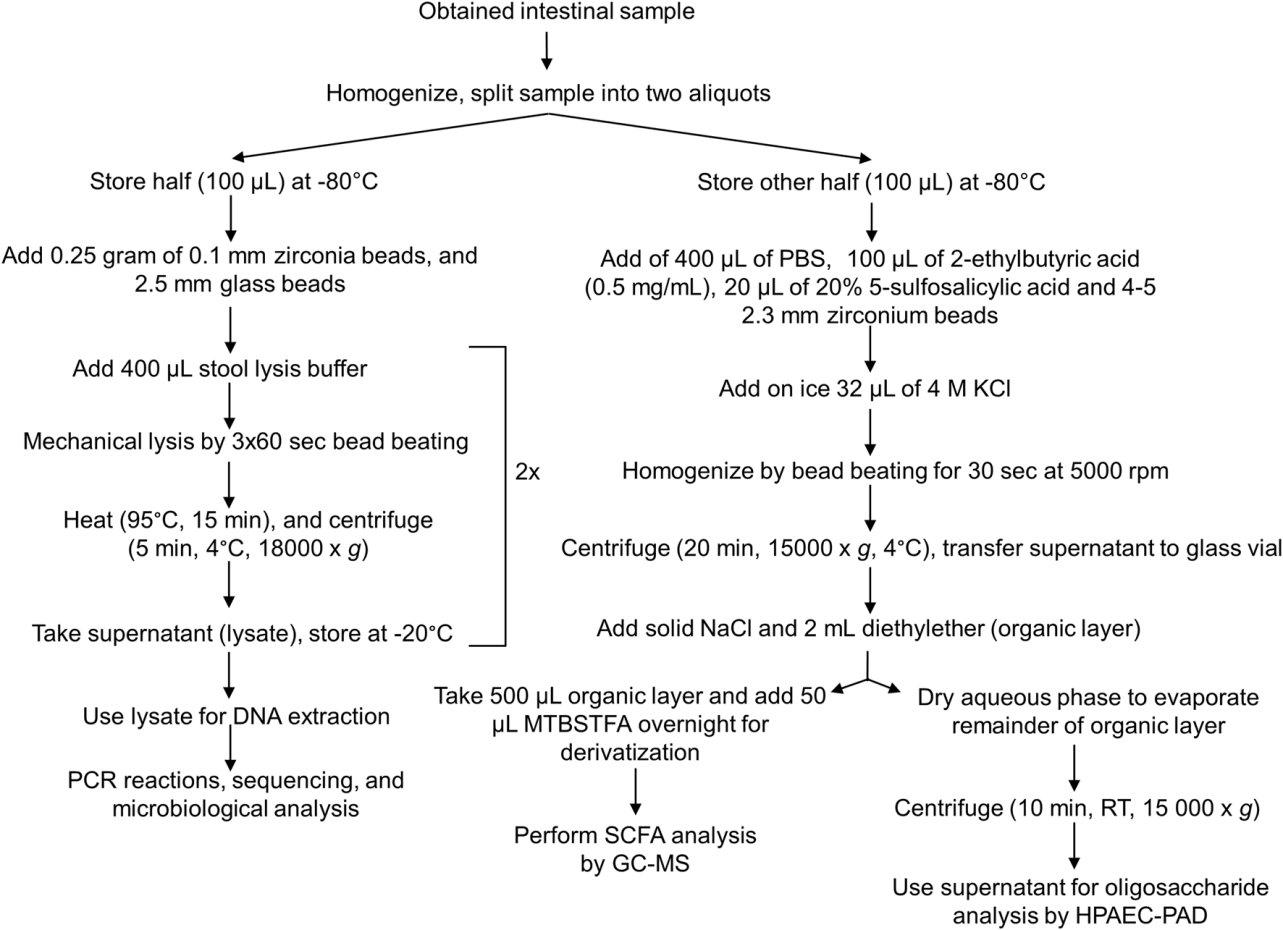
A schematic overview of the complete protocol to measure oligosaccharides, SCFA, and microbiota composition. *GC–MS* gas chromatography-mass spectrometry, *HPAEC-PAD* high performance anion exchange chromatography with pulsed amperometric detection, *SCFA* short chain fatty acids.

We then tested if omission of HCl would affect the extraction of SCFA. Without HCl addition, but still in the presence of sulfosalicylic acid, the pH in the aqueous layer was 1.7, well below the pK*a* of the SCFA. Consequently, the SCFA concentrations measured in standard curves (Fig. 5F–H) and in faeces samples (Fig. 5I) were very similar. Apparently, under these conditions, recovery of SCFA did not depend on the addition of HCl. In conclusion, SCFA extraction without HCl allows the measurements of SCFA and also soluble fibres without splitting the sample. The final combined protocol to investigate all primary outcomes is shown in Fig. 6.

## Discussion

In humans the consumption of dietary fibres has been linked to beneficial health effects^1,2^. Detailed knowledge on the fate of fibres inside the human intestinal tract is however still very limited, due to the lack of convenient non-invasive methods to study the fate of dietary fibres inside the digestive tract in vivo. New gastrointestinal sampling capsules are being developed, which sample luminal content at a specific location in the intestinal lumen^11,14,15^. Application of these devices pose several methodological challenges, because of their retrieval time delay and their small volume. In this study we addressed these challenges and developed a toolbox for stabilising the sample by a quenching reagent and analysing the fermentation status of dietary fibres in such samples, i.e. the extent of fibre degradation, the microbiota composition, and fermentation products.

### The developed quenching reagent

Using this novel technology to obtain intestinal luminal samples, there is the challenge of obtaining a representative sample because of the considerable time delay between sampling and retrieving the sample. For that purpose, we developed a quenching reagent and tested its effectiveness to inhibit fibre breakdown in vitro in human ileostomy and faecal samples. The quenching reagent was based on a bacterial lysis buffer with addition of several components to denature enzymes. This reagent can be preloaded in gastrointestinal capsules to block further fermentation of fibres in the obtained sample. The components in the quenching reagent (NaCl, EDTA, Tris, SDS, urea, at pH 8.5) were present for specific reasons: NaCl was needed to establish ionic strength outside the cells, EDTA is a chelating agent that complexes enzyme metal-cofactors and thereby blocks DNAses, and Tris is a buffering agent to maintain the pH around 8.5 for DNA stability. The anionic detergent SDS was added for disruption of bacterial cell membrane structures^43^ and to inhibit nuclease activity, thereby preventing DNA degradation. Urea (8 M) was added as a general protein denaturant^34^. The quenching reagent showed efficient quenching of dietary fibre fermentation in vitro for up to 48 h.

Importantly, the developed methodology was evaluated in faecal samples from different donors. Blocking fibre breakdown could be donor and consequently microbiota dependent, due to differences in microbial capacity of degrading non-digestible carbohydrates. Faecal samples are a more challenging matrix than small intestine samples, and better represent the large intestine where most of the fermentation occurs. As is clear from this study, the quenching reagent stopped degradation effectively in all faecal samples, irrespective of the fibre tested or microbial composition present. The concentrations of SCFA measured in the samples in this study before the start of fermentation were lower than typically measured in faeces^44^, because the faecal samples had been diluted in SIEM with dietary fibres to prepare the faecal slurry.

The microbiota composition can differ between in vitro fermentation samples and the in vivo situation in humans, since in vitro only a viable fraction will persist. It is noteworthy that different faecal donors and fibre mixes produced strikingly different SCFA patterns. Considering these different breakdown patterns by different compositions of microbiota, we developed an effective quenching reagent for all individuals, and since it contains general enzyme inhibitors and components for cell lysing, we expect that all microbial activity is stopped when added, preserving the sample for a broader range of metabolites. Furthermore, we showed that our quenching reagent was effective over a period of 48 h, which we expect to be the maximum intestinal transit time in vivo*.* Previously, the human whole gut transit time was shown to be 30.6 ± 7.7 h^12^. In this way, we ensure that the sample obtained is a representative sample of the sampling location.

### Toxicological report of components in the quenching reagent

Gastrointestinal sampling capsules have a reservoir opening towards the gastrointestinal lumen. Although leaking of quenching reagent into the human intestinal lumen is very unlikely, the quenching reagent should be safe for human oral intake. A safety risk assessment focussed on oral acute toxicity of the components in the quenching reagent was performed. For this assessment it was assumed that the lumen would be exposed to the total volume of quenching reagent present in the capsule reservoir, which is 50 µL. An overview of the quenching reagent component concentrations versus present toxicology information is given in Table 1.

**Table 1:** Toxicology information about the components in the quenching reagent.

Chemical name	CAS registration number	Concentration	Amount mg/50 µl	Toxicology information
Trisaminomethane, thrometamine (Tris)	77-86-1	175 mM	1.1	Oral (non-human) NOAEL = 4000 mg/kg
Sodium chloride (NaCl)	7647-14-5	530 mM	1.5	GRAS component
Ethylenediaminetetraacetic acid (EDTA)	60-00-4	35 mM	0.7	EFSA, no safety concern (humans) = 1.9 mg/kg/day
Sodium dodecylsulfate, sodium laurylsulfate (SDS)	151-21-3	12%	6	Oral (non-human) NOAEL = 100 mg/kg/day
Urea	57-13-6	8 M	24	GRAS component

The concentrations of all components and amounts of the components present in 50 µL of the quenching reagent, the CAS registration numbers, and information about toxicology is presented.

*CAS* chemical abstracts service, *GRAS* generally recognized as safe, *NOAEL* no observed adverse effect level, *EFSA* European Food Safety Authority.

Trisaminomethane (Tris) is also known as pharmaceutical registered under the name THAM, and is a biological buffer agent that regulates acid–base regulation^45, 46^. The so-called ‘no observed adverse effect level’ (NOAEL) for repeated oral intake of Tris is 4000 mg/kg body weight^47^. Therefore, a single dose of 1.1 mg Tris in the quenching agent is not considered as being harmful. SDS is on the FDA list of multipurpose additives allowed to be directly and indirectly added to food^48^. Adult consumers may be exposed to up to 0.030 mg SDS/kg body weight/ day, and the NOAEL was established for repeated dose toxicity being 100 mg SDS/kg body weight/day. The amount of SDS in our quenching reagent (6 mg) is therefore not considered to be harmful for an adult subject. The other components sodium chloride (NaCl), urea, and (disodium) EDTA are generally recognized as safe food substances by the FDA^49,50^. Therefore, the presence of small amounts in this quenching reagent are not of concern. In conclusion, based on literature research none of the quenching reagent components that are present in 50 μL will lead to acute toxicity effects in humans, and can therefore be considered as safe.

### The toolbox for combined analysis of small samples

Another challenge of the technology for intestinal sampling, is the small volume of sample that can be obtained. Therefore, to retrieve as much information as possible from a single small sample, analytical protocols were optimized for the measurement of fibres, microbiota composition and SCFA in a small sample in the presence of the quenching reagent. We started from the assumption that the expected maximal volume to be retrieved from a sampling capsule will be around 200 µL^14^. In the final protocol after homogenization the sample was therefore divided in two aliquots of 100 µL: one for SCFA and fibres, and the other for microbiota analysis. For complete recovery of bacterial DNA from the sample, the sample needed to be divided into two aliquots first, since the quenching reagent partially lysed the cells, leading to the presence of bacterial DNA in the supernatant. This prevented recovery of bacterial DNA from the pellet only, and therefore a separate aliquot of the intact sample was used. For SCFA analysis, precipitation of SDS using KCl was found to be crucial to measure correct SCFA amounts in a sample in the presence of the quenching reagent. Perturbations of the analysis of fibres were minimal. Only retention times shifted due to the high salt content. Analysis of the fibre standard in the presence of high salt content corrected for this problem sufficiently. When using our protocol to measure SCFA using GC with HCl acidification of the samples, and the fibres of interest were hydrolysed to their respective mono- and disaccharides. Therefore, HCl was omitted while maintaining 5-sulfosalicylic acid for protein precipitation. Apparently, the pH of the sample decreased enough to recover SCFA and avoid fibre and oligosaccharide hydrolysis. This optimizations of the combined analytical protocols enabled us to use only 100 µL to measure both SCFA and fibres. This combined protocol is also applicable and relevant for other research fields, where researchers have to deal, for other reasons, with small sample volumes. Moreover, we identified the main sources of disturbance to be tested in another analytical assays, in order to expand the toolbox to study other interesting microbial processes.

## Conclusions

We developed and validated a toolbox that can be used to obtain and analyse a representative sample of intestinal content using novel gastrointestinal sampling capsules. The quenching reagent presented, can completely block fibres fermentation and SCFA production for up to 48 h. Furthermore, a mixed protocol was developed to measure fibres, bacterial DNA and SCFA from a small sample in the presence of the quenching reagent. This work is the basis for a more extensive analytical approach to also study other gut microbial processes. Considering the small volumes of samples expected to be obtained and the cost of novel gastrointestinal sampling capsules, the developed toolbox will be a major advantage in this rapidly developing research field.

## Supplementary Information

Supplementary Information.

## Data Availability

All data generated or analysed during this study are included in this published article and its supplementary information files.

## Abbreviations

ANOVA: Analysis of variance
ASL: Stool lysis buffer
ASV: Amplicon sequence variants
BSTFA: N,O-Bis(trimethylsilyl)trifluoroacetamide
CV: Coefficient of variation
DNA: Deoxyribonucleic acid
dNTPs: Deoxynucleoside triphosphate
DP: Degree of polymerization
FDA: Food and Drug Administration
FOS: Fructo-oligosaccharides
GC–MS: Gas chromatography–mass spectrometry
GOS: Galacto-oligosaccharides
HCl: Hydrochloric acid
HPAEC: High performance anion exchange chromatography
KCl: Potassium chloride
MTBSTFA: N-tert-butyldimethylsilyl-N-methyltrifluoroacetamide
NaCl: Sodium chloride
NOAEL: No-observed-adverse-effect level
PAD: Pulsed amperometric detection
PBS: Phosphate-buffered saline
PCR: Polymerase chain reaction
rRNA: Ribosomal ribonucleic acid
SCFA: Short chain fatty acids
SDS: Sodium dodecylsulfate
SI: Small intestine
SIEM: Standard ileal efflux medium
TBS: Tert-butyldimethylsilyl
THAM: Tris-hydroxymethyl aminomethane
