# Supplementary material for: A toolbox for the comprehensive analysis of small volume human intestinal samples that can be used with gastrointestinal sampling capsules
Source: Sci Rep. 2021 Apr 14;11:8133. doi: 10.1038/s41598-021-86980-y (PMC8046781; doi:10.1038/s41598-021-86980-y)
Supplement: Supplementary file 1 — Supplementary Information. [file 41598_2021_86980_MOESM1_ESM.docx]

**Title page**

**Title:** A toolbox for the comprehensive analysis of small volume human intestinal samples that can be used with gastrointestinal sampling capsules.

**Authors:** Melany Rios-Morales^*^ (m.y.rios.morales@umcg.nl), Mara P.H. van Trijp^*^ (mara.vantrijp@wur.nl), Christiane Rösch (christianeroesch@gmx.de), Ran An (ran.an@wur.nl), Theo Boer (t.boer@umcg.nl), Albert Gerding ([a.gerding@umcg.nl](mailto:a.gerding@umcg.nl)) , Naomi de Ruiter (naomi_de_ruiter@hotmail.com), Martijn Koehorst ([m.koehorst@umcg.nl](mailto:m.koehorst@umcg.nl)), M. Rebecca Heiner-Fokkema (m.r.heiner@umcg.nl), Henk A. Schols (henk.schols@wur.nl), Dirk-Jan Reijngoud^1^ (d.j.reijngoud01@umcg.nl), Guido J.E.J. Hooiveld^#^ (guido.hooiveld@wur.nl), Barbara M. Bakker^#^ (b.m.bakker01@umcg.nl)

**Supplementary Methods**

**Quenching reagent development:** During quenching reagent development, the inhibiting potential of selected quenching components based on literature research was tested on fibre substrates incubated with commercially available fibre degrading enzymes (Supplementary Table 1). Standard mixtures of GOS (2.5 mg/mL), and chicory FOS/inulin (2.5 mg/mL) were prepared in MES buffer (25 mM, pH 5.8). For the incubations, 100 µL of GOS was incubated with 15 µL of β-galactosidase (EC 3.2.1.23) ^1^ (10 mg/mL) isolated from Aspergillus oryzae (Lactase DS-K, Amano Enzyme Inc., Japan), with either 100 µL control (water) or potential quenching reagent components. Furthermore, 100 µL of FOS/inulin was incubated with 2 µL endo-inulinase (EC 3.2.1.7) isolated from Aspergillus niger ^2^ (Novozym 960, Novozymes A/S, Denmark), with either 100 µL control (water) or potential quenching reagent components. Incubations took place for 16 hours at 37°C.

**Supplementary Results**

**Quenching reagent development:** Known metal ion inhibitors of β-galactosidase and inulinases (Supplementary Table 1), were tested ^3-5^. Ag^+^ and the combination Ag^+^/Cu^2+^ inhibited β-galactosidase, since there was 90% GOS 1<DP>7 or 96% GOS 1<DP>7 left after incubation, respectively. Cu^2+^ did not inhibit GOS breakdown. Ag^+^, Cu^2+^ and Zn^2+^ only partial inhibited exo- and endo-inulinases, compared to water incubations. A protein denaturing formulation (PDF) was tested as a more general enzyme inhibitor ^6^ (Supplementary Table 1). PDF did not inhibit β-galactosidases (4% GOS 1<DP>7), compared to control (0.5% GOS 1<DP>7), nor inulinases (15.8% FOS/inulin 11<DP>20) compared to control (0% FOS/inulin 11<DP>20). The components in the PDF interfered with HPAEC-PAD analysis. Moreover, a RNA conserving reagent ^7^ was tested (Supplementary Table 1), but it did not inhibit inulinases (346% DP=1 formed) compared to control (318% DP=1 formed). Overall, both the PDF and the RNA conserving reagent were not tested in more detail.

**Considerations after sample retrieval:** When samples were retrieved at the end of the incubations, reactions in the presence of quenching reagent should be stopped by freezing at ‑80°C, and not by heat inactivation, unlike what is sometimes described in literature ^8^. When fermentation samples were heat inactivated in the presence of quenching reagent, FOS DP<7 disappeared from the sample (Supplementary Fig. S1), likely due to a reaction between saccharides and urea during heating.

**Analytical methods validation of SCFA:** The measurement of SCFA by GC-MS in human faecal samples was validated. After collection, faeces were stored at 4°C, homogenized using a paint shaker and afterwards stored at -80°C. For SCFA analysis, there are two pre-analytical steps: extraction and derivatisation. Standard curves and samples should always be prepared using the same PBS volume, due to a putative partitioning effect between the aqueous and organic solvent phase ratio. Bead beating of faeces led to a more effective extraction of SCFA eliminating any matrix effect, since after the standard addition of known SCFA concentrations to the same faecal sample slope of the standard curve remained the same (Supplementary Fig. S3A-D). Afterwards, SCFAs were extracted into the diethyl-ether layer and derivatised by silylation ^9^, resulting in tert-butyldimethylsilyl (TBS/TBDMS) derivatives, which are highly volatile, less polar, and more thermostable ^9^. During the entire pre-analytical process, the bead beating and all centrifugation and vortex steps were performed at 4°C. Ten aliquots of the same faeces were prepared at the same time, and SCFA were measured in the same GC-MS sequence. The intra-assay variation of acetate, propionate, and butyrate of coefficient of variation (CV) was <10% (Supplementary Fig. S3E). The ten aliquots of the same faeces sample were measured at 10 different times with an inter-assay variation of acetate, propionate, and butyrate CV <15%, without carry-over or changes in GC-MS injection repeatability (Supplementary Fig. S3E). For 100 mg faeces the final validated protocol is described in the Methodology.


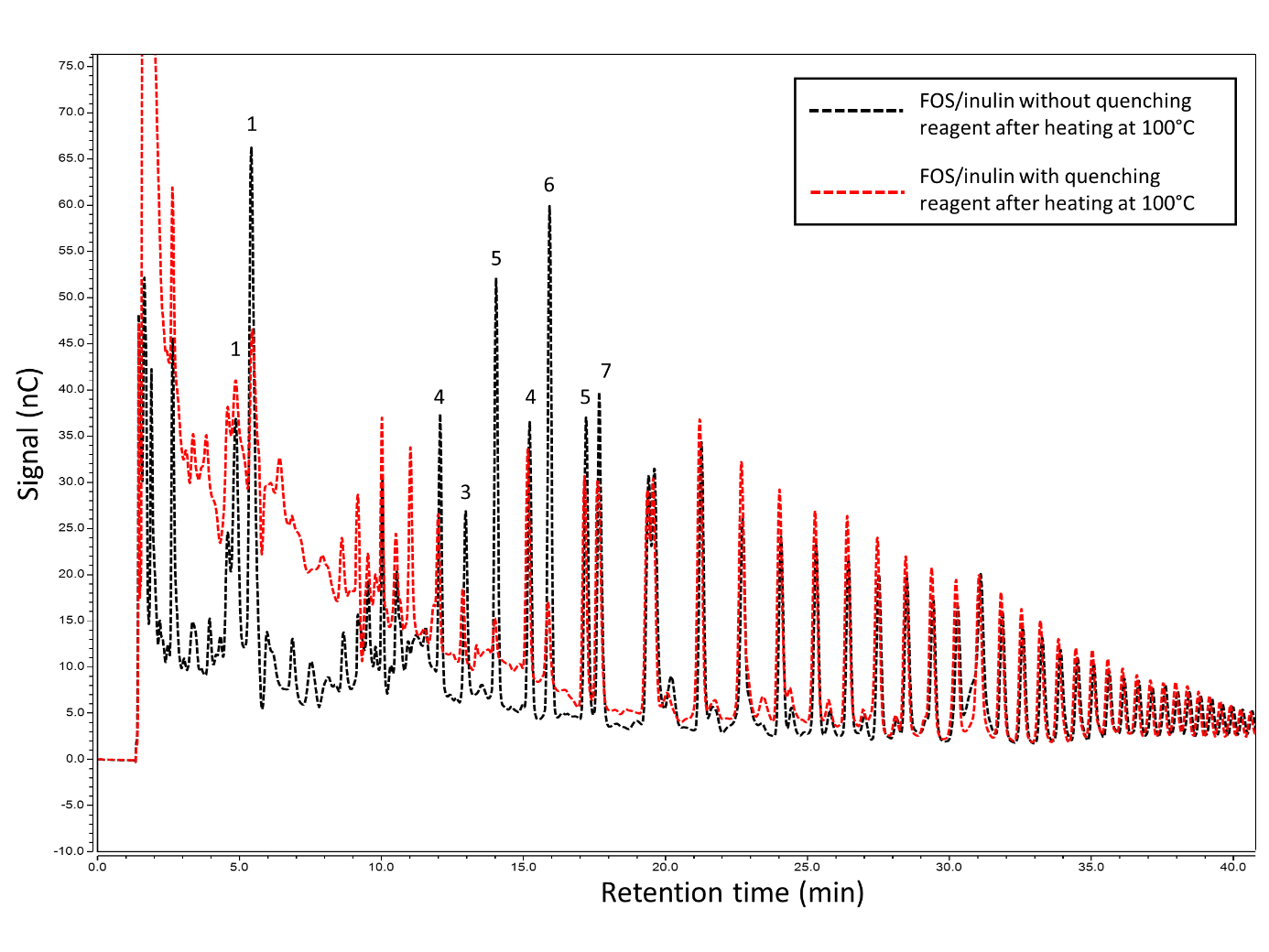
**Supplementary Fig. S1.** **The effect of heating on one small intestine chicory FOS/inulin fermentation sample with and without quenching reagent***.* The black chromatogram represents FOS/inulin without added quenching reagent after heating at 100°C, the red chromatogram represents FOS/inulin present in the same sample with added quenching reagent after heating at 100°C. The numbers in the chromatogram represent the degree of polymerization, 1 = monomers, 2 = dimers, ≥3 = oligomers.

**Supplementary Table S1. The quenching liquids and their components that were tested for quenching chicory FOS/inulin and GOS in the presence of inulinases or galactosidase, respectively.**

| **Quenching liquid** | **Concentrations of components in stock solution** | | **Remaining  chicory FOS/inulin (area %)** | | | **Remaining GOS (area %)** | |
| --- | --- | --- | --- | --- | --- | --- | --- |
|  | | | **DP=1** | **1<DP≤11** | **11<DP>20** | **DP=1** | **1<DP>7** |
| **Metal ions** | | | | | | | |
| Control | Water | | 943.6 | 7.29 | 0 | 357.0 | 0.516 |
| Ag^+^ | 5 mM | | 50.9 | 44.3 | 36.6 | 187.2 | 90.76 |
| Cu^2+^ | 10 mM | | 161.5 | 46.3 | 23.3 | 347.6 | 13.34 |
| Zn^2+^ | 10 mM | | 161.9 | 114.0 | 31.95 | N.A. | N.A. |
| Cu^2+^, Ag^+^ | 5 mM each | | 393.5 | 27.16 | 26.0 | 154.9 | 96.24 |
| **RNA conserving reagent** | | | | | | | |
| Control | Water | | 318.3 | 109.6 | 5.36 | N.A. | N.A. |
| RNA conserving reagent | Sodium citrate (25 mM) EDTA (10 mM) Ammonium sulphate (50 gram/100 mL) | | 346.4 | 100.8 | 73.15 | N.A. | N.A. |
| **Protein denaturing formulation** | | | | | | | |
| Control | | Water | 943.6 | 7.29 | 0 | 357.0 | 0.516 |
| Protein denaturing formulation (PDF) | | Ethanol (40% v/v) Lithium chloride (3.5 M) Sodium citrate (50 mM) | 162.0 | 58.3 | 15.8 | 414.4 | 3.97 |

The values represent the area under the peaks after incubation compared to the area under the peaks before incubation (area %). Fibres profiles were obtained with HPAEC-PAD as described in Materials and Method, and peak area was quantified per component up to the maximum degree of polymerization (DP) measured by HPAEC-PAD which is dependent on the structure of the fibre. N.A. is not analyzed.

**Supplementary Table S2.** **The bacterial lysis buffer combinations that were tested for quenching chicory FOS/inulin and GOS in the presence of inulinases or galactosidase, respectively.**

| **Quenching liquid** | **Concentrations of components in stock solution** | **pH** | **Remaining  chicory FOS/inulin (area %)** | | | **Remaining GOS (area %)** | |
| --- | --- | --- | --- | --- | --- | --- | --- |
|  |  |  | **DP=1** | **1<DP≤11** | **11<DP>20** | **DP=1** | **1<DP>7** |
| **Bacterial lysis buffer combinations** | | | | | | | |
| Control | Water | **-** | 1233 | 39 | 0.3 | 284 | 0.4 |
| Bacterial lysis buffer with Urea | Tris (50 mM) NaCl (150 mM) EDTA (10 mM) SDS (1.5%) Urea (8 M) | 6.5 | 286 | 86 | 71 | 262 | 24 |
|  |  | 8.5 | 201 | 127 | 131 | 146 | 60 |
|  |  | 9.5 | 136 | 88 | 72 | 129 | 95 |
| Bacterial lysis buffer with Urea, proteinase K | Tris (50 mM) NaCl (150 mM) EDTA (10 mM) SDS (1.5%) Urea (8 M) Proteinase K (300 µg/mL) | 8.5 | 221 | 135 | 140 | 141 | 71 |
| Bacterial lysis buffer with Urea, proteinase K, Ag^+^ | Tris (50 mM) NaCl (150 mM) EDTA (10 mM) SDS (1.5%) Urea (8 M) Proteinase K (300 µg/mL) Ag^+^ (20 mM) | 8.5 | 154 | 106 | 100 | 89 | 78 |

The values represent the area under the peaks after incubation compared to the area under the peaks before incubation (area %). Fibres profiles were obtained with HPAEC-PAD as described in Materials and Method, and peak area was quantified per component up to the maximum degree of polymerization (DP) measured by HPAEC-PAD which is dependent on the structure of the fibre.

**Supplementary Fig. S2.** **Quenching reagent development, effectiveness of blocking breakdown of GOS and chicory FOS/inulin.** The inhibitory capacity of the quenching liquid stock solution (pH 8.5, 50 mM Tris, 150 mM NaCl, 10 mM EDTA, 1.5% SDS, 8 M urea) on degradation of GOS (A, C) and FOS/inulin (B, D) by commercially available galactosidase and inulinases with additions of proteinase K (300 µg/mL) or additions of proteinase K and Ag^+^ (20 mM) (A, B), also tested at different pH values (C, D). The area under the peaks of the HPAEC-PAD chromatograms of mono- di and oligosaccharides after 16 h incubation is presented (the maximum degree of polymerization, DP, is dependent on the structure of the fibre). The peak area is given in nanocoulomb (nC)*retention time in minutes. No error bars are shown. DP; degree of polymerization, FOS; fructo-oligosaccharides, GOS; galacto-oligosaccharides, prot. K; proteinase K.


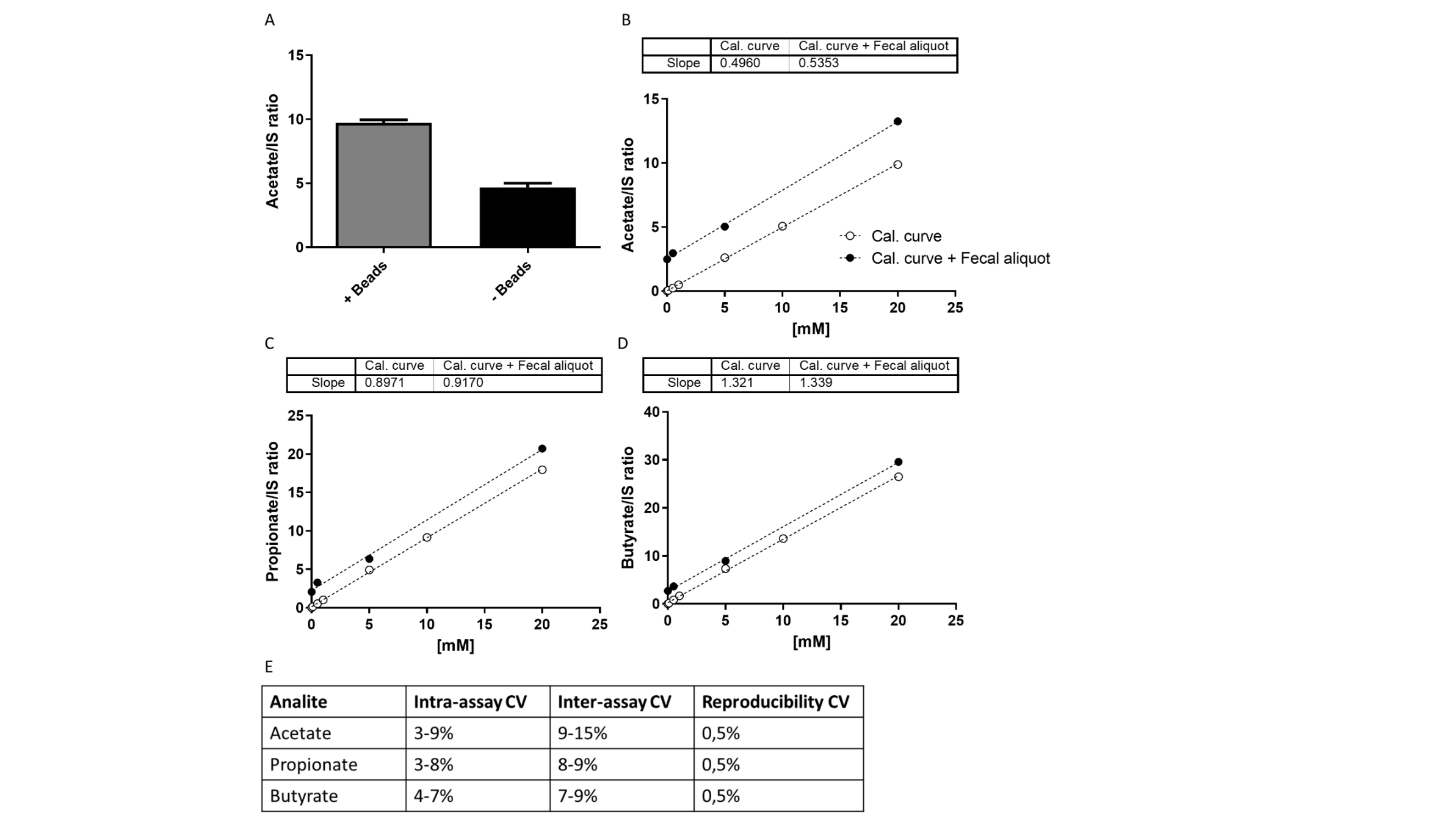


**Supplementary Fig. S3.** **SCFA protocol validation using human faecal samples.** (A) Acetate/internal standard ratio after extraction with and without bead beating homogenization, (B,C,D) Standard addition of acetate, propionate, and butyrate respectively after performing the protocol as described in Materials and Methods including the bead beating homogenization, (E) Intra-assay, inter-assay and reproducibility coefficients of variation % of acetate, propionate, and butyrate. CV, coefficient of variation.


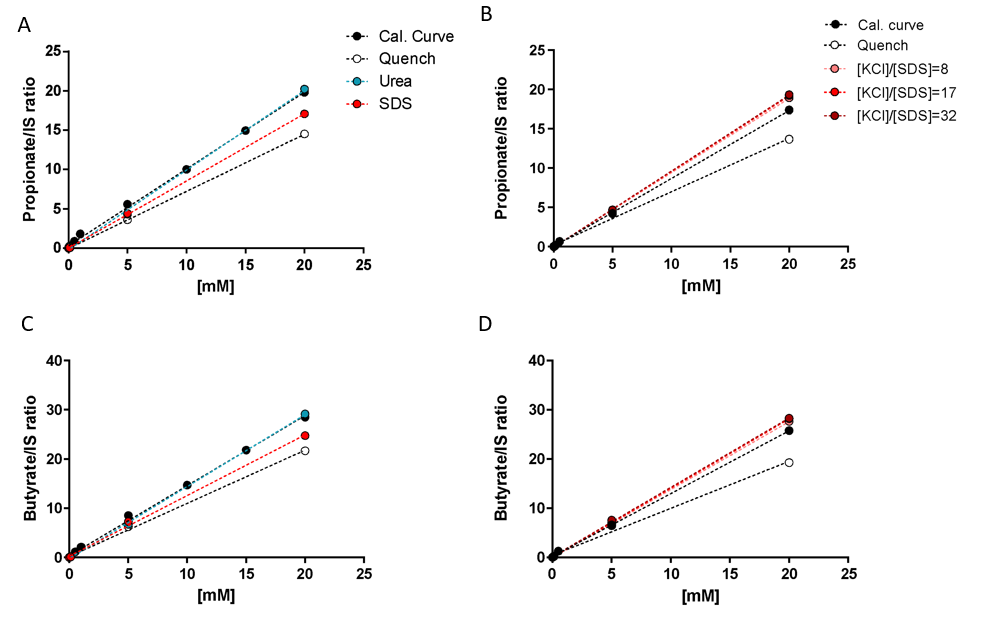
**Supplementary Fig. S4. Quenching reagent interference in SCFAs analysis by GC-MS.** (A, C) Calibration curves of propionate and butyrate made in PBS with known concentrations (0-20 mM) in the presence of the quenching reagent or its major components Urea and SDS, (B,D) Calibration curves of propionate and butyrate with SDS precipitation by KCl in different KCl/SDS molar ratios.

**Supplementary Table S3.** **The peak areas and total peak area of the chromatograms from FOS and GOS standards in water with or without quenching reagent and with and without the addition of KCl.**

| **Sample** | **Peak area (nC*min)** | |  |  |  |  |  |  |  |  |  |  |  |  | **Total peak area (nC*min)** | **% total peak area of fibres mixtures in water** |
| --- | --- | --- | --- | --- | --- | --- | --- | --- | --- | --- | --- | --- | --- | --- | --- | --- |
| **FOS** | **Peak 1 (DP2)** | **Peak 2 (DP3)** | **Peak 3 (DP2)** | **Peak 4 (DP4)** | **Peak 5 (DP3)** | **Peak 6 (DP5)** | **Peak 7 (DP4)** | **Peak 8 (DP6)** | **Peak 9 (DP5)** | **Peak 10 (DP7)** | **Peak 11 (DP6)** | **Peak 12 (DP8)** |  |  |  |  |
| in water | 5.2 | 3.9 | 1.9 | 8.3 | 33.3 | 11.3 | 22.5 | 9.3 | 10.9 | 3.8 | 6.9 | 1.7 |  |  | 119.1 | - |
| in water with quenching reagent | 5.1 | 3.7 | 1.9 | 8.1 | 32.2 | 10.9 | 21.8 | 9.0 | 10.6 | 3.8 | 6.8 | 1.7 |  |  | 115.6 | 97.1 |
| in water with quenching reagent and with KCl | 5.2 | 3.7 | 1.9 | 8.1 | 31.9 | 10.6 | 21.1 | 10.7 | 10.1 | 4.6 | 6.7 | 1.7 |  |  | 116.4 | 97.7 |
| **GOS** | **Peak 1 (DP1)** | **Peak 2 (DP3)** | **Peak 3 (DP2)** | **Peak 4 (DP2)** | **Peak 5 (DP3)** | **Peak 6 (DP2)** | **Peak 7 (DP3/4)** | **Peak 8 (DP3)** | **Peak 9 (DP4)** | **Peak 10 (DP4)** | **Peak 11 (DP4)** | **Peak 12 (DP5)** | **Peak 13 (DP5)** | **Peak 14 (DP6)** |  |  |
| in water | 14.9 | 2.4 | 1.6 | 44.8 | 8.6 | 36.6 | 31.5 | 13.2 | 6.0 | 7.8 | 4.8 | 4.4 | 1.7 | 1.2 | 186.0 | - |
| in water with quenching reagent | 15.2 | 0.9 | 1.3 | 45.1 | 9.8 | 36.8 | 31.4 | 13.1 | 5.8 | 7.8 | 4.7 | 4.3 | 1.6 | 1.2 | 182.6 | 98.2 |
| in water with quenching reagent and with KCl | 14.3 | 1.8 | 1.3 | 45.5 | 8.7 | 36.9 | 31.3 | 13.2 | 5.7 | 8.5 | 4.8 | 4.5 | 1.7 | 1.5 | 183.8 | 98.8 |

The total peak area was quantified from the HPAEC-PAD chromatograms. For FOS, the peaks and the total peak area between retention time 6 and 23 minutes were quantified. For GOS, the peaks and the total peak area between 3.7 and 17 minutes were quantified. The area is given in nanoCoulomb multiplied by the retention time in minutes (nC ⋅ min). The peak area was subsequently expressed as a % of the peak area of fibre in water (without quenching reagent and without KCl). FOS, fructo-oligosaccharides; GOS, galacto-oligosaccharides.

**References**

1 Tavares, T. M., F.X. in *Encyclopedia of food sciences and nutrition (second edition)* (eds B. Caballero, P. Finglas, & F. Toldra) 6157-6163 (Academic Press, 2003).

2 Sirisansaneeyakul, S., Worawuthiyanan, N., Vanichsriratana, W., Srinophakun, P. & Chisti, Y. Production of fructose from inulin using mixed inulinases from aspergillus niger and candida guilliermondii. *World J. Microbiol. Biotechnol.* **23**, 543-552 (2007).

3 Uchiyama, T., Miyazaki, K. & Yaoi, K. Characterization of a novel β-glucosidase from a compost microbial metagenome with strong transglycosylation activity. *J. Biol. Chem.*, jbc. M113. 471342 (2013).

4 Dey, P. M. Inhibition, transgalactosylation and mechanism of action of sweet almond α-galactosidase. *Enzymology.* **191**, 644-652 (1969).

5 Chi, Z., Chi, Z., Zhang, T., Liu, G. & Yue, L. Inulinase-expressing microorganisms and applications of inulinases. *Appl. Microbiol. Biotechnol.* **82**, 211-220 (2009).

6 Paulsen, K. E. Formulations and methods for denaturing proteins. U.S. patent (2003-12-16).

7 Lader, E. S. Methods and reagents for preserving rna in cell and tissue samples. U.S. patent (2001-03-20).

8 Ahern, T. & Klibanov, A. The mechanisms of irreversible enzyme inactivation at 100c. *Science.* **228**, 1280-1284 (1985).

9 Primec, M., Mičetić-Turk, D. & Langerholc, T. Analysis of short-chain fatty acids in human feces: A scoping review. *Anal. Biochem.* **526**, 9-21 (2017).
